# Predicting Composite Component Behavior Using Element Level Crashworthiness Tests, Finite Element Analysis and Automated Parametric Identification

**DOI:** 10.3390/ma13204501

**Published:** 2020-10-11

**Authors:** Ravin Garg, Iman Babaei, Davide Salvatore Paolino, Lorenzo Vigna, Lucio Cascone, Andrea Calzolari, Giuseppe Galizia, Giovanni Belingardi

**Affiliations:** 1 Department of Mechanical and Aerospace Engineering, Politecnico di Torino, Corso Duca degli Abruzzi 24, 10129 Turin, Italy; iman.babaei@polito.it (I.B.); davide.paolino@polito.it (D.S.P.); lorenzo_vigna@polito.it (L.V.); giovanni.belingardi@polito.it (G.B.); 2 Polymers and Glass Department, Group Materials Labs, Centro Ricerche Fiat, Pomigliano d’Arco, 80038 Naples, Italy; lucio.cascone@crf.it; 3 Illinois Tool Works Inc. (ITW) Test and Measurement Italy, Instron Compagnia Europea Apparecchi Scientifici Torino (CEAST), Via Airauda 12, Pianezza, 10044 Turin, Italy; Andrea_Calzolari@instron.com (A.C.); giuseppe_galizia@instron.com (G.G.)

**Keywords:** crashworthiness, impact behavior prediction, automated parametric identification, composite materials, finite element analysis

## Abstract

Fibre reinforced plastics have tailorable and superior mechanical characteristics compared to metals and can be used to construct relevant components such as primary crash structures for automobiles. However, the absence of standardized methodologies to predict component level damage has led to their underutilization as compared to their metallic counterparts, which are used extensively to manufacture primary crash structures. This paper presents a methodology that uses crashworthiness results from in-plane impact tests, conducted on carbon-fibre reinforced epoxy flat plates, to tune the related material card in Radioss using two different parametric identification techniques: global and adaptive response search methods. The resulting virtual material model was then successfully validated by comparing the crushing behavior with results obtained from experiments that were conducted by impacting a Formula SAE (Society of Automotive Engineers) crash box. Use of automated identification techniques significantly reduces the development time of composite crash structures, whilst the predictive capability reduces the need for component level tests, thereby making the development process more efficient, automated and economical, thereby reducing the cost of development using composite materials. This in turn promotes the development of vehicles that meet safety standards with lower mass and noxious gas emissions.

## 1. Introduction

In 2012, the European Union decided to reduce vehicular average emissions by 27% from 2015 to 2021 and, using 2021 as a baseline, further reduce them by 15% and 37.5% by 2025 and 2030, respectively [1]. Around the same time, US regulators published new Corporate Average Fuel Economy regulations that dictated increasing average fuel economy to 54.5 mpg for cars and light-duty trucks by 2025 [2]. Automakers responded by not only investing in downsizing and electrifying their powertrains, but also in optimizing assemblies and components, as evidenced by an increase in these research topics [3,4,5]. Component optimization led to an increased use of composite materials in vehicles due to the potential advantages they offered, such as improved impact resistance, reduced noise and vibrations, improved integration in assemblies with fewer subcomponents, etc., in addition to weight savings [6,7,8]. 

The improved specific energy absorption capabilities of structures made from composite materials, as compared to those made from metals, have been well-documented [9,10,11,12]. This improvement is because composite materials absorb energy through a variety of failure modes such as delamination, fragmentation, buckling, fibre breakage and matrix cracking while crushing progressively [13,14,15]. Despite their potential, composite materials have not replaced metals for use as primary crash components due to the time and cost of development and lack of predictive modelling capability of their damage behavior [16]. In order to develop components made of composites, extensive testing needs to be undertaken at all levels of the building block approach (BBA): coupons, component, assembly and full-scale testing, which can prove to be expensive and time consuming [17]. Use of advanced numerical tools can help reduce these monetary and time costs by aiding prediction of component, assembly, and full-scale behavior [11]. To aid prediction quality, material cards need to be calibrated using a trial-and-error approach at an element level [18,19,20,21,22,23] or calculated using analytical models based on experimental data available [24]. Trial-and-error approaches are time consuming and it is not always possible to obtain all the data required for analytical models. Data, if obtained, come at the cost of an extensive experimental campaign, which again requires time and monetary resources. Therefore, there arises a need for an identification procedure that automatically calibrates the material card to be used in tests that involve composite components. Identification studies, thus far, have not been completely automated as they combine a trial-and-error approach with a response surface approximation and calibrate the parameters only to a defined set of mean crush forces and not the entire force curve [14,20,25]. 

**Figure 1:**
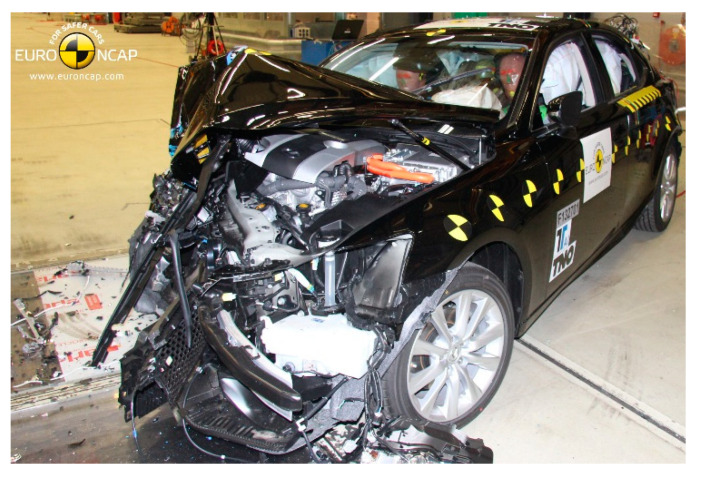
Damage after frontal crash test conducted by Euro New Car Assessment Program [27].

The present study summarizes a methodology that can be used to predict component level damage behavior using numerical models that have been tuned using results of axially impacted flat composite plates, with calibration of the same conducted using parametric identification, all of which was done using the HyperWorks software package. HyperMesh was used for pre-processing, HyperView and HyperGraph for post-processing, HyperStudy for parametric identification and Radioss as a solver. Macro and meso-scale approaches have been considered to develop procedures suitable for industrial structure design. In order to test the flat plates, the methodology takes advantage of an anti-buckling fixture developed by Politecnico di Torino and Instron [26]. Flat composite carbon fibre reinforced polymer (CFRP) specimens were impact tested in the in-plane direction using the fixture. Force-displacement curves, obtained from the experimental procedure, were used to calibrate the material card in Radioss, which modelled the experimental procedure to replicate similar behaviors using the considered macro and meso-scale approaches. The macro-scale approach involved modelling the composite plates using only shell elements, which could not capture delamination, whilst the meso-scale approach modelled the plate using both shell and cohesive elements that enable delamination and frond formation. In order to avoid the cumbersome trial-and-error approach for the calibration of the material card parameters, parametric identification was performed on three numerical failure parameters available in the implemented material model, parameters that cannot be obtained directly from experiments. This methodology constitutes a repeatable procedure, easily applicable to different structures or materials. The calibrated card was then used to predict the damage and validate the force-displacement curves using results from a Formula SAE impact attenuator manufactured from the same material and tested up to a 9700 J impact. It was impacted at various velocities using a six-meter-high drop tower in a manner similar to that seen in real word automotive crashes, as shown in Figure 1. 

The paper presents the experimental and numerical methodology and the automated identification setup in Section 2, results of the experimental and numerical campaign and its discussion in Section 3 and conclusions in Section 4.

## 2. Materials and Methods

### 2.1. Material Characterization and Experimental Setup

For the first level of the BBA, standard ASTM tests were performed for tensile (D3039), compression (D3410), and flexural (D0790) characterization of the CFRP laminates made of GG630T-37 [28] 2 × 2-twill high strength carbon quasi-isotropic fabric, results of which are presented in Table 1. 

**Figure 2:**
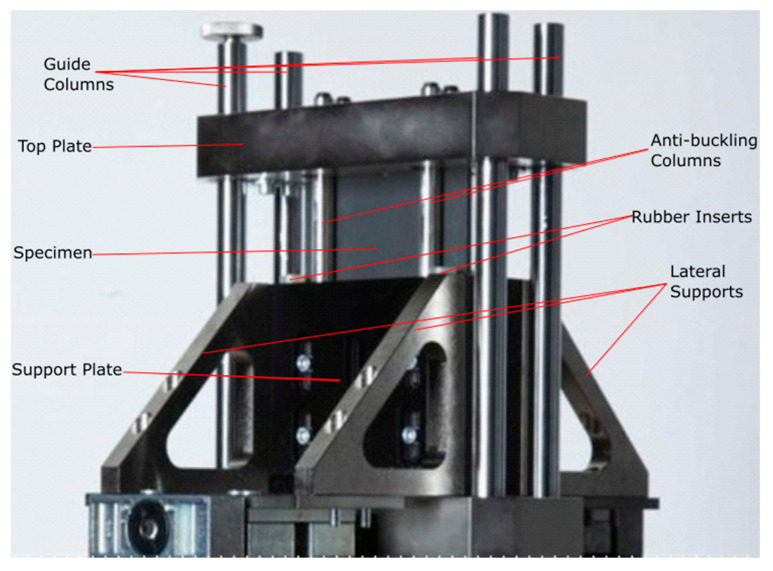
New anti-buckling fixture designed for crashworthiness evaluation of flat composite plates under axial impact load [26].

**Figure 3:**
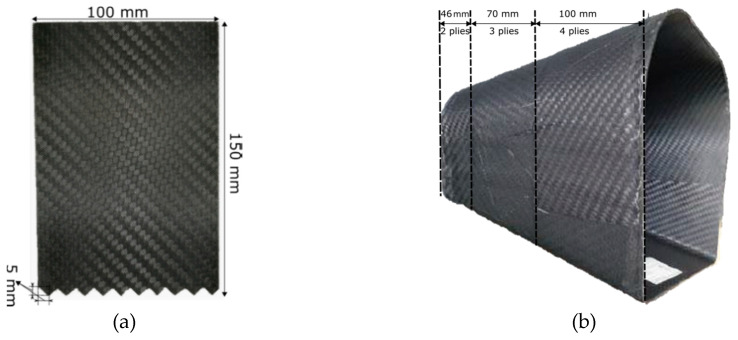
(**a**) Flat specimen with saw tooth triggers for element level tests; (**b**) crash attenuator with varying thickness to initiate steady crushing under impact during component level tests.

Then, moving upward in the BBA, in-plane impact tests were executed, using Instron 9450 drop tower testing machine (Instron, Pianezza TO, Italy,) with 1800 J of impact energy capacity, on saw-tooth triggered flat specimens to evaluate the energy absorption capabilities in the element level. Since flat elements tend to buckle under in-plane forces, a new anti-buckling fixture was designed and manufactured for these tests [26]. Figure 2 shows an image of this fixture and Figure 3a shows the triggered flat specimen used for this test. 

For the final step of the BBA, six crash attenuators were manufactured to analyze their responses under different impact loading conditions. The attenuators were composed of three sections with different wall thicknesses, which were intended to work as trigger mechanisms, ensuring that steady crushing started from the top of the specimens. Figure 3b shows an image of the attenuator with the heights and number of plies for the three different sections.

At first, two quasi-static tests at 10 mm/min were performed using ZwickRoell electromechanical testing machine (ZwickRoell, Kennesaw, GA, US) with 100 kN of load capacity. Then, four different dynamic tests with 300 kg impact mass were carried out using the drop weight testing facility at Picchio Spa (Ancarano, Teramo, Italy). Table 2 mentions the impact velocities and the corresponding impact energies. The tower facility had a maximum drop height of 6 m and could provide up to 26 kJ impact energy. MMF_KD38V piezoelectric accelerometer (Metra Mess- und Frequenztechnik, Radebeul, Germany) was used for data acquisition and high-speed camera with a capture speed of 1000 frames per second were put in the place to capture the displacement data and enable tracking of the crushing procedure.

### 2.2. Numerical Modelling

The flat-plate model was composed of a composite material specimen impacted by a falling steel plate, while the composite plate sat on a steel base and was held in place by four anti-buckling supports [26]. Metallic components, such as the impactor, anti-buckling supports and base, were modelled using Johnson–Cook elastoplastic material model. All these components were modelled using C40 steel properties obtained from the Total Materia database. The properties are mentioned in Table 3 [29]. Simulations were conducted using six out of eight available cores of Intel i7 CPU @ 2.70 Hz with 16 GB RAM (HP ZBook 15 G3, Made in China).

**Figure 4:**
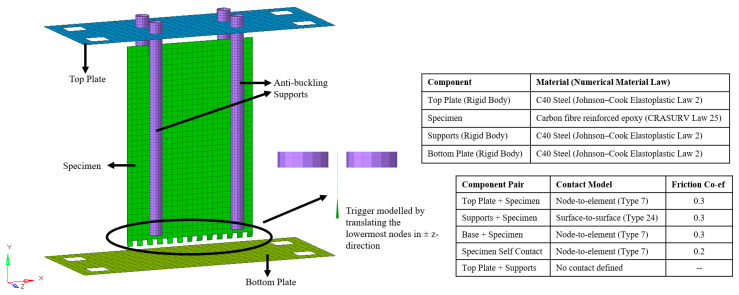
Overview of the flat-plate model and the relevant material and contact modelling information.

Composite material, CFRP in this case, was modelled using the CRASURV formulation of material law 25 in Radioss. Table 4 reports the material properties of the CFRP composite. Mechanical properties obtained from characterization tests were validated numerically using one-element tests conducted in tension, compression, and shear. The laminate was 2.56 mm thick and 150 × 100 mm in length and width. It was composed of four plies, each of which was 0.64 mm thick. Since a woven quasi-isotropic fabric material was used to manufacture the laminate, properties in 1 and 2 directions were assumed equal. Figure 4 reports the respective materials and laws used.

CRASURV formulation is a modified form of the Tsai-Wu criteria that was developed as a part of the IMT 3 Brite Euram EU program and was validated on various composite materials and the underbelly and airframe of the A320 aircraft, and has since been used extensively in the aerospace industry [30,31]. CRASURV allows failure and hardening (for shear in case of CFRP) parameters to be defined in both 1 and 2 directions for tension, compression and shear and is, therefore, more robust as compared to Tsai-Wu criteria which does not allow this bifurcation for different directions and load cases [25]. Softening was modelled as a linear reduction in the stress after ultimate strength was reached until the residual stress for compression and shear. Residual stresses were constant until failure. For tension, no residual stress was inputted, and softening occurred until 120% of ultimate strain, the default value set by the solver, after which the element would be deleted. Failure could be modelled as energy based, wherein a limit value could be defined for the energy absorbed, and/or strain based, wherein the limit value could be defined as the maximum allowable strain. Deletion occurred according to the model chosen. If both the models were chosen, deletion was dependent on the event that occurred first. The mathematical formulation of the failure surface is shown in Equation (1) and shows how it is different as compared to the classical Tsai-Wu formulation, wherein the $F_{ij}$ factors are functions of only the respective first damage stresses and not of the respective damage work per unit volume, $W_{p,ij}$. CRASURV formulation also models these $F_{ij}$ factors as functions of the strain rate, but strain rate effects were not accounted for this study as the experimental investigation, conducted between 4–8 m/s, showed that strain rate effects were negligible within this velocity range.

(1) $$F_{1}\left(W_{p,1}\right)\sigma_{1}+F_{2}\left(W_{p,2}\right)\sigma_{2}+F_{11}\left(W_{p,1}\right)\sigma_{1}^{2}+F_{22}\left(W_{p,2}\right)\sigma_{2}^{2}+F_{44}\left(W_{p,12}\right)\sigma_{12}^{2}+2F_{12}\left(W_{p}\right)\sigma_{1}\sigma_{2}<1$$

where F is the variable coefficient, $W_{p}$ is the damaging work per unit volume, σ is the stress in material coordinate system, and 1, 2 are the principal directions [32]. 

Property type 11, a property available in Radioss for a composite shell modelling, was used to define the layup of the CFRP laminate. It allowed for modelling the element type, thickness, layer position and orthotropic direction of each ply. Property type 1, i.e., simple shell, was used to model metallic components. Fully integrated Batoz shell elements were used for all the models. Contact modelling was defined using the node-to-element (Type 7) and surface-to-surface (Type 24) contacts. Respective contact models used are reported in Figure 4. Minimum contact stiffness of 1 kN/mm was applied in order to avoid a very soft contact, which would be unrealistic. Friction coefficients between CFRP and steel and amongst CFRP elements were obtained from the literature [33,34,35]. Thickness changes in the components were accounted for through changes in contact stiffness in accordance with Equation (2). No maximum contact stiffness was inputted. Contact stiffnesses of master ($K_{m}$) and slave ($K_{s}$) elements were calculated using Equations (4) and (5).

(2) $$K=\max\left[St_{min},\;min\left(St_{max},K_{0}\right)\right]$$

(3) $$K_{0}=\min\left(K_{m},K_{s}\right)$$

(4) $$K_{m}=\mathrm{Stfac}\times 0.5\times E_{m}\times t_{m}$$

(5) $$K_{s}=\mathrm{Stfac}\times E_{s}\times t_{s}$$

where K is the element contact stiffness, $St_{min}$ is the minimum contact stiffness, $St_{max}$ is the maximum contact stiffness, Stfac is the stiffness factor that scaled the contact stiffness, E is the Young’s modulus, t is the thickness, and s and m are slave and master elements, respectively.

Anti-buckling cylindrical supports were modelled using a 1 mm mesh to capture the column curvature, while base and impactor plates were modelled with a 5 mm mesh size. All three components were modelled as rigid bodies. Boundary conditions applied to the base and anti-buckling supports resulted in zero degrees of freedom (DoFs) for these components, whilst for the impactor a single DoF was set, allowing it to translate freely in the y-direction (falling and rebounding) only. Composite specimen was modelled with 4 node quadrilateral (quad) shells, with a 4 mm mesh size, which was considered the best trade-off between accuracy and efficiency, based on previous published studies conducted on crashworthiness of composite structures [20,36,37]. As the objective of the study was to simulate and predict the experimental test on a Formula SAE crash box in order to validate the methodology, a 4 mm mesh size avoided any significant increases in the number of elements required to model the component and proportional increase in the DoFs.

The plate trigger was machined as a sawtooth for experiments as shown in Figure 3a. In order to model the same, triangular (tria) elements would need to be used. Since these elements behave stiffer than quad elements, considering that the trigger is machined to initiate progressive crush, the trigger was modelled with quad elements. Modelling the trigger with quad elements allowed the specimen to be modelled with a structured mesh. In addition, the lowermost nodes of the trigger were translated 0.25 mm in the z-direction (transverse to the plate), half in the positive z-direction and the other half in the negative z-direction, in order to initiate progressive crushing. This ensured a softer failure initialization wherein subsequent rows of elements were not deleted resulting in troughs bottoming at zero force when seen from force-displacement plots. The final model consisted of approximately 12,500 elements and 6800 DoFs and is depicted in Figure 4. As some components had nodes that were not free to translate or rotate in all directions due to the boundary conditions applied, the residual DoFs were less than the total number of elements in the model. The composite specimen component was made of 940 elements and 6100 DoFs.

A similar modelling approach was followed for modelling the impact attenuator. The model consisted of the CFRP attenuator placed between a steel steady base and steel falling plate. Metallic components were modelled with a 10 mm mesh size, whilst the attenuator was modelled with a 4 mm mesh size to ensure continuity from the flat-plate methodology as impact behavior is mesh dependent. Owing to the geometry of the attenuator, it was composed of both quad and triangular elements. The attenuator was divided into three sections: top section made of two plies 46 mm long, middle section made of three plies 70 mm long and bottom section made of four plies 100 mm long, wherein each ply was 0.75 mm thick.

**Figure 5:**
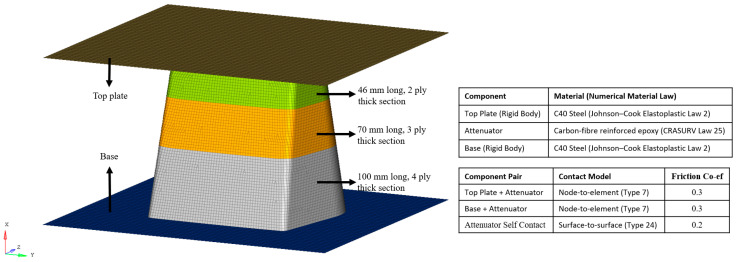
Impact attenuator model with relevant material and contact modelling information.

The material card was unchanged from the flat-plate model, to ensure continuity regarding the material properties. Property card was modified to account for the different ply configurations. Base and top plates were made rigid bodies to ensure all elements moved synchronously. Boundary conditions applied to the base ensured no DoFs, whilst those applied to the falling plate allowed it to translate in the x-direction (vertical) only. The final model consisted of approximately 14,000 elements and 64,500 DoFs, while the attenuator was composed of 62 tria and 10,300 quad elements that allowed 62,700 DoFs. The model is shown in Figure 5.

### 2.3. Numerical Modelling with Cohesive Elements

As delamination modelling, which is a major failure mode in impact cases [13,38,39], was not possible with shell elements, both flat plate and impact attenuator were also modelled using an appropriate mix of shell and cohesive elements. Cohesive elements were only applied to the composite components; therefore, there was no change in the model with respect to the metallic components or contact modelling. 

The composite flat plate was composed of four plates made of shell elements, each of which represented a ply 0.64 mm thick. The three solid cohesive element layers, modelled between the four plies, were modelled as 0.64 mm thick in order to fill the gap between the shells. The final model was composed of 18,100 element and 25,000 DoFs, of which the composite specimen was composed of 6500 elements and 24,300 DoFs. Law 59 Connect was used to model the adhesive layer, which constituted the cohesive elements. This law allowed elastic and inelastic behavior to be modelled in normal and shear directions [32]. Table 5 reports the material properties of the adhesive layer, which were obtained from published studies on epoxy material as no characterization tests were conducted on the epoxy material used [40,41,42].

The adopted failure criterion was strain-based. It was preferred over the energy-based, as an energy-based criterion requires data from double cantilever beam (DCB) and end-notched flexure (ENF) tests, which were not available [24,43,44]. DCB and ENF tests are used to obtain mode I and mode II fracture toughness, respectively, of composite materials. Fracture toughness governs the adhesion between plies of composite laminate, as it is the material’s resistance to crack propagation. As nodes of cohesive elements were merged with those of shell elements, there was no need to add an interface between the cohesive elements and the plies as the behavior of cohesive elements was completely dependent on the behavior of the corresponding shell elements. 

**Figure 6:**
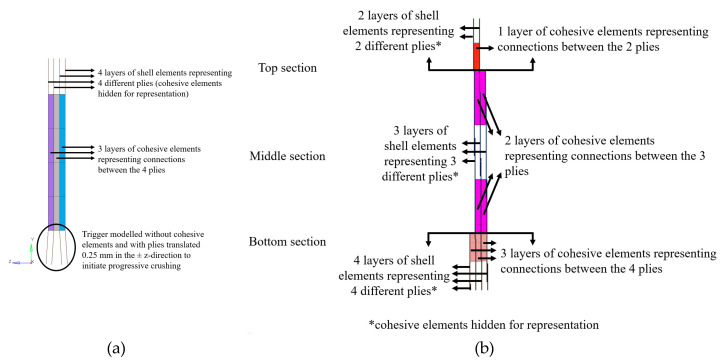
(**a**) Cross section of the flat plate model with cohesive elements and each ply represented by separate shell elements (**b**) Cross section of the impact attenuator model with cohesive elements and each ply represented by separate shell elements.

A similar approach was followed for the impact attenuator: the top section was composed of a layer of cohesive elements bounded by two layers of shell elements, the middle section was composed of two layers of cohesive elements bounded by three layers of shell elements, and the bottom section was composed of three layers of cohesive elements bounded by four layers of shell elements. The only difference between the flat plate and attenuator modelling using cohesive elements was the thickness of the plies and the cohesive elements. The thickness used for the impact attenuator was 0.75 mm. Each layer of cohesive elements represented adhesion between the plies and each layer of shell elements represented a ply. The final model was made of 62,000 elements and 211,000 DoFs, of which the attenuator was made of 58,000 elements and 209,000 DoFs. Figure 6 shows cross-section views of both the flat plates and the impact attenuator. 

### 2.4. Automated Identification Setup

HyperStudy was used for identification runs due to the ease of its interface with Radioss. The objective of the identification was to set a more robust procedure as compared to the trial-and-error approach used in previous published studies in order to tune the material cards, thereby reducing the setup time. Use of identification for calibration also eliminated the subjectivity involved in the trial-and-error approach and the possibility that the calibrated values thus obtained were not the global minimum. The general setup of the identification involved defining the input variables and their respective bounds, running a system bounds check to ensure that the analysis did not give any errors when the lower and upper bounds were used, defining and evaluating an output response, setting an objective for the output response, choosing the optimization approach to follow, running the identification and post-processing, in that order.

The numerical parameters that could be optimized were:

- $W_{p\_max}$: Global maximum damaging work per unit volume
- $W_{pmax\_c1}$: Compressive maximum damaging work per unit volume in 1 direction
- $\sigma_{res\_c1}$: Compressive residual stress in 1 direction
- $W_{pmax\_c2}$: Compressive maximum damaging work per unit volume in 2 direction
- $\sigma_{res\_c2}$: Compressive residual stress in 2 direction
- $W_{pmax\_t12}$: Shear maximum plastic damaging per unit volume in 12 direction
- $\tau_{res\_t12}$: Shear residual stress in 12 direction

Damaging work was the limit value in the element deletion criteria while residual stresses were used to define softening behavior. As for the particular type of material considered in this study, properties were assumed to be the same in 1 and 2 directions; therefore, $\sigma_{res\_c1}=\;\sigma_{res\_c2}$ and $W_{pmax\_c1}=\;W_{pmax\_c2}$. Failure criterion deleted the element depending on the minimum of the global and failure mode specific values; therefore, only $W_{p\_max}$ was considered sufficient. This resulted in the need for optimizing just the following three parameters: global maximum damaging work per unit volume ($W_{p\_max}$), compressive residual stress ($\sigma_{res}$) and shear residual stress ($\tau_{res\_t12}$). Upper bounds for the residual stresses were fixed as the ultimate strength (Table 4) and the lower bounds were fixed as 10% of the upper bound. Upper bound for $W_{p\_max}$ was fixed as 0.12 J/mm^3^, based on a value obtained from the literature [45], while the lower bound as 0.008 J/mm^3^, which was the area under the shear stress–strain curve.

**Figure 7:**
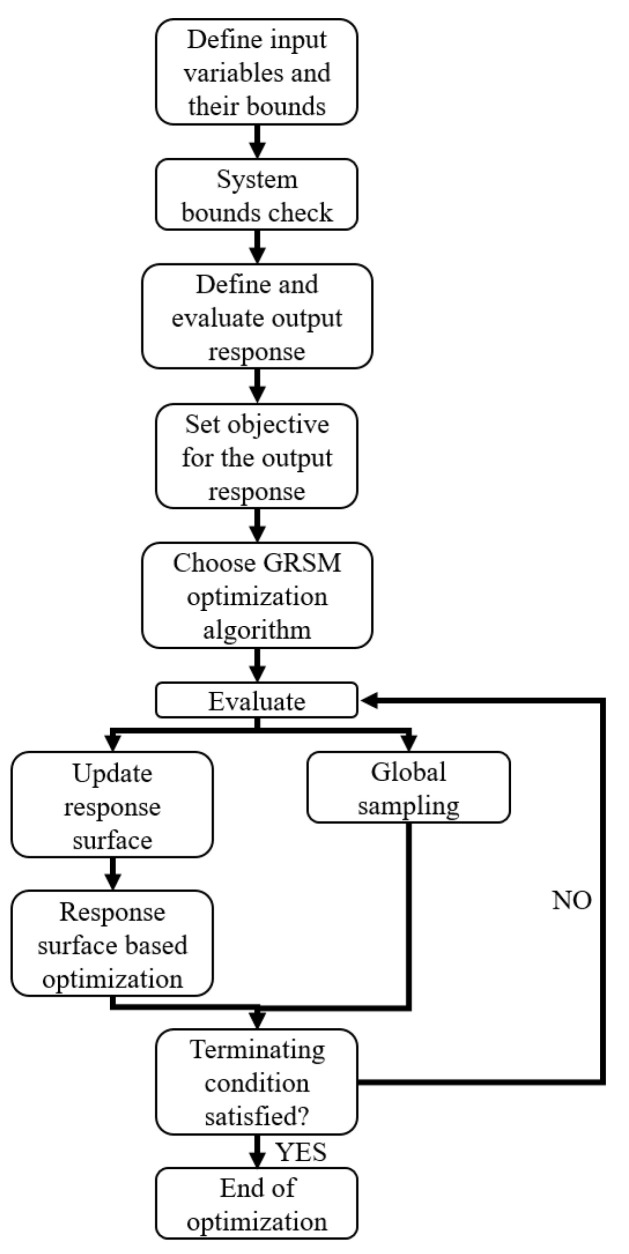
Different phases of the global response search method (GRSM) identification.

Of the various optimization algorithms available in HyperStudy, global response search method (GRSM) and adaptive response search method (ARSM) were selected due to their suitability to the problem: deterministic single objective optimization with multiple variables and few constraints. The response surface for both GRSM and ARSM were updated after each iteration. It is known from the literature that GRSM works better when a global optimum is desired and there are a number of design variables. In addition, GRSM is known to be more robust as compared to ARSM, whereas the latter is more efficient [46,47]. Based on the numerical tests conducted both GRSM and ARSM were able to predict the mean crush force and stroke displacement accurately. ARSM arrived at the final solution in 23 iterations, whilst GRSM took 50 iterations, therefore, confirming that ARSM is more efficient. GRSM, however, gradually converged to the global minimum, whereas ARSM appeared more as a randomized solution. Thus, GRSM appears to be more robust as compared to ARSM. GRSM, due to its robustness, was selected to develop the methodology, while ARSM could be an alternative in industrial applications where efficiency is paramount and safety factors are the norm when using composite materials. Figure 7 shows a flowchart detailing the process of the GRSM identification.

Two response functions were tested: the integral of absolute difference between the experimental and the simulation values of the crush force as shown in Equation (6) and the integral of squared difference between the experimental and the simulation values of the crush force as shown in Equation (7). Although both the methods produced a material card that lead to close correlation between the experimental and numerical average crushing force, the displacement stroke was better modelled with the absolute difference approach as compared to the squared difference approach. The absolute difference approach led to a final difference of 7% while the squared difference approach led to a final difference of 25% as compared to the experimental stroke displacement. Hence, the absolute difference approach was preferred and used for subsequent calculations.

(6) $$Response\;Objective_{abs}=minimize\left[\int_{t=0}^{\infty }\left|F_{exp}-F_{sim}\right|\;dt\right]$$

(7) $$Response\;Objective_{sq}=minimize\left[\int_{t=0}^{\infty }{\left(F_{exp}-F_{sim}\right)}^{2}\;dt\right]$$

The identification setup for flat plate with cohesive elements was similar to that of the runs with only shell except that the upper bound for $W_{p}$ was increased to 0.6 J/mm^3^ as the upper bound used for identification with shell element model only were too low leading to the optimized value arriving at the upper bound.

## 3. Results and Discussion

Tests on triggered flat specimens were used to calculate the specific energy absorption (SEA) of the material. To calculate SEA, the following formula was used:

(8) $$SEA=\;\frac{E}{\rho A\delta }=\;\frac{\int Fdx}{\rho A\delta }$$

where E is the absorbed energy, F is the crush force, ρ is the material density, A is the plate cross section, and δ is the length of the crushed part. Force-displacement results obtained from element level tests were used for these calculations by performing trapezoidal numerical integration of the data. Table 6 shows the SEA values for tests performed on this level at different impact velocities. No significant effect of a change in mass was observed on the SEA values indicating that they were insensitive to a change in mass. Impact energy was absorbed by splaying of the outer layers and fragmentation of the inner ones. This is in close accordance with reported energy absorption mechanisms of composites in the literature [48,49]. A 1.5 mm displacement of the base plate, not accounted for in the force-displacement results, was observed during post-processing when viewed from the high-speed camera.

**Figure 8:**
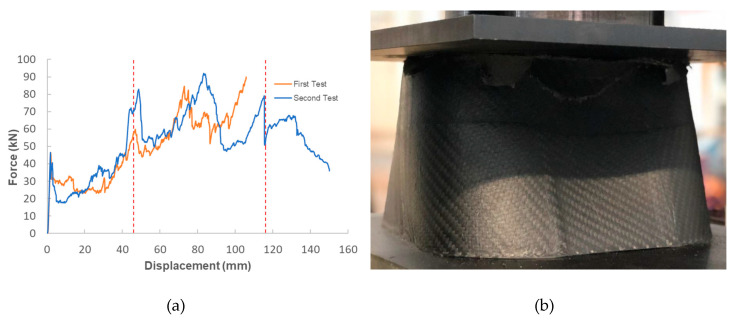
(**a**) Force displacement curves for the tests performed under quasi-static conditions; (**b**) Composite component being tested under quasi-static conditions with ZwickRoell electromechanical testing machine.

For the impact attenuator, two quasi-static tests were performed for better understanding of the material and demonstrator response under crush forces. Figure 8a shows the force displacement results of these tests and Figure 8b shows the component during the compression test done with an electromechanical testing machine. Vertical lines in Figure 8a signify the interface between the sections of the attenuator. The apparatus used allowed either 150 mm of displacement or 100 kN of force. For test one, after a displacement of 105 mm buckling of the walls was significant and unrelated to the quasi-static crushing of the attenuator. Hence, the test was stopped. For test two, a similar behavior was observed at 110 mm, however, buckling was immediately followed by a break in the attenuator which led to a subsequent drop in force. 

**Figure 9:**
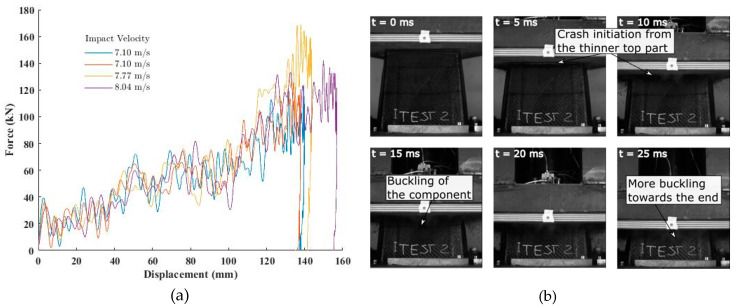
(**a**) Force displacement results of the dynamic tests at three different impact velocities, (**b**) Images of the crash attenuator during impact at different times captured by high-speed camera: impact velocity = 7.10 m/s, impact mass = 300 kg, and impact energy = 7561 J.

Finally, four dynamic impact tests were performed on the remaining four impact attenuators. Figure 9 shows the results of the dynamic tests and a series of photos extracted from the high-speed capture of the experiment at different times showing crash initiation and buckling of the front and back walls as the experiment progressed. As there was not a significant change in the load-displacement curves with the change in the impact velocities, no strain rate effects were observed. Furthermore, it should be noted that the range of velocities tested was very narrow, 4–8 m/s. Three of the final displacements were in the 135–145 mm range, while the last test, conducted with a higher impact velocity (8.04 m/s) resulted in a slightly larger value of the final displacement (155 mm). A 6–8 mm displacement of the base, not accounted for in the force-displacement results, was observed during post-processing when viewed from the high-speed camera. All the four graphs overlapped each other, further showing that strain rate effects were negligible. The high oscillation in the raw data acquired with the sampling frequency of 50 kHz was filtered out with Channel Frequency Class (CFC) filter in accordance with ISO 6487 and J211/1_201403 standards regarding the instrumentation for impacts tests of road vehicles [50,51].

In order to study the effect of the contact stiffness between components during contact modelling, all contacts were initially set to the minimum stiffness value of 1 kN/mm. Seven combinations of contact stiffnesses were studied as mentioned in Table 7 for the flat plate impact tests. The solver, according to Equation (2), calculated the respective stiffnesses. Activation of the calculation for the contact stiffness between the anti-buckling columns and the specimen did not have an effect on the results as is evidenced by no change in deformation type between cases A and B, D and F and E and H because it was only a sliding contact. However, the activation of the calculation for the contact stiffness between the base, impactor and specimen and between the elements of the specimen did affect final damage. Fronding at the bottom as observed in experiments and was seen in cases D, E, F and H. Identifications were conducted on cases E and F to arrive at the best fit values for the variables mentioned in Section 2.2. Since results of case D were the same as F and those of case H were the same as E, cases D and H were not considered for the identification procedure, as their counterparts were more encompassing. Additionally, also case G was considered for identification, as it was possible that the identification led to better results.

**Figure 10:**
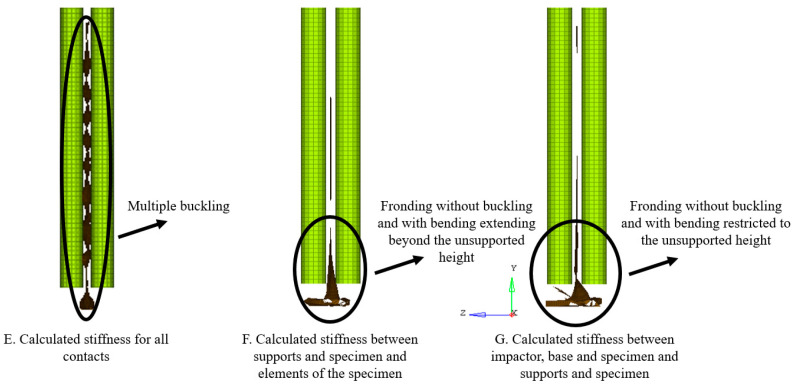
Comparison of optimized results of three contact stiffness formulation cases; labels are according to Table 7.

Of the three cases considered in the identification process, E led to multiple buckling in the entire length of the specimen possibly because the contacts became too stiff. Cases F and G, correctly, developed fronds only at the plate bottom, but for case F frond formation extended above the unsupported height while for case G it was restricted only below the unsupported height. Therefore, contact stiffness calculation based on Young’s modulus and thickness was activated for the contacts between the impactor, base and specimen and the specimen and the supports while a stiffness of 1kN/mm was used for the interface between the elements of the specimen. Case G was the final identification run on the flat-plate and Figure 10 shows the final deformation of the same. As expected, fronding as observed in experiments could not be seen as clearly as delamination modelling was not incorporated due to the use of a single shell that represented all plies.

**Figure 11:**
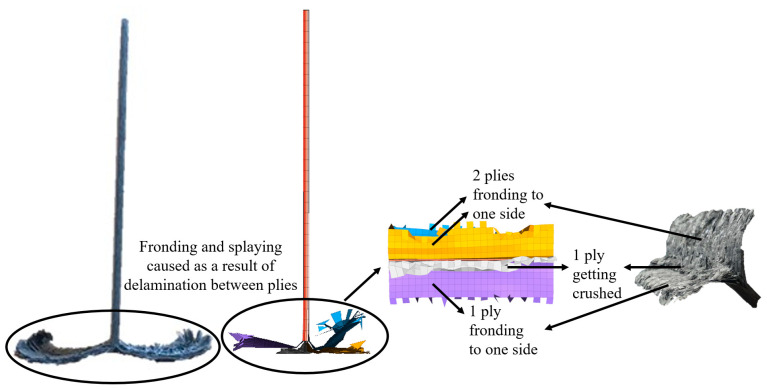
Damage visualization of shell and cohesive element flat plate model upon a 550 J impact at 7 m/s.

Fronding, as observed in experiments, could be seen clearly, when cohesive elements were introduced into the model as shown in Figure 11.

**Figure 12:**
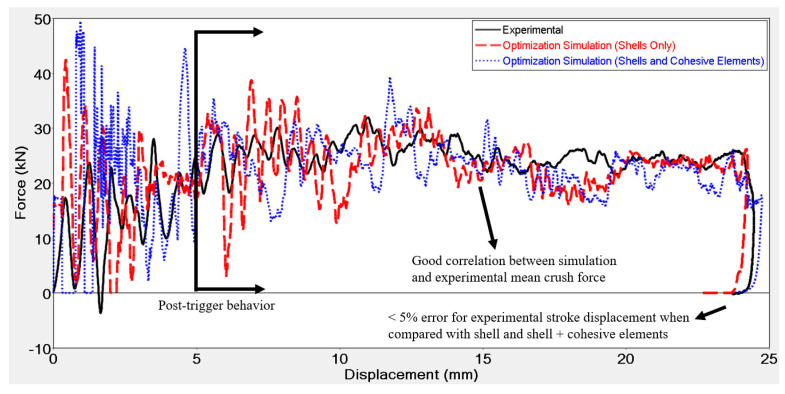
Force vs. displacement comparison of experimental and optimized simulation of a CFRP flat plate subjected to a 550 J impact with an impact velocity of 7 m/s using both shell and shell + cohesive elements.

Results of the optimized iterations derived from final identification runs conducted on flat plates using both the macro and meso-scale approach at 550 J impact energy and 7 m/s impact velocity are shown in Figure 12.

Upon visual inspection of the experimental tests, it was observed that two plies fronded to one side, while one fronded to the other and one of the middle plies was crushed. A similar behavior was observed in the simulation as evidenced in Figure 11. 

Identifications were conducted with GRSM algorithm and absolute difference was used as the response function. The identification consisted of 50 iterations leading to a total run time of about 250–300 min, as each iteration was 5 min long for macro-scale approach. Run time with the meso-scale approach was 65 min for each iteration resulting in a total run time for the identification of about 60 h. Identification simulations were able to converge to a material card that could model the crushing force after initial part that was influenced (disturbed by) the trigger with reasonable accuracy (see Figure 12). Stroke displacement for the model with shell elements only was within 5% of difference with respect to the experimental results, whilst that for the model with shell and cohesive elements was equal to the experimental results. If the 1.5 mm displacement caused due to flexion of the base in the experiments was subtracted from the final displacement of the experiments, the error in stroke displacement obtained from the shell element model reduced further, whilst that for the model with shell and cohesive elements would be < 5%. In the trigger region, both models overestimate the force initially, before converging to the experimental results. The deviation from experimental results in the trigger region was because the trigger was not modelled exactly as it actually was in the physical flat plate. Modelling the trigger as sawtooth could improve the correlation between numerical and experimental results. However, this would be at the expense of using an unstructured mesh. Additionally, the trigger was 5 mm in length in the physical flat plate and a mesh size of 4 mm permitted only one and a quarter element to cover the trigger region. Using a finer mesh in this region could improve the correlation between experimental and numerical results, whilst avoiding the use of an unstructured mesh. Best fit parameters for failure energy and residual stresses are reported in Table 8.

**Figure 13:**
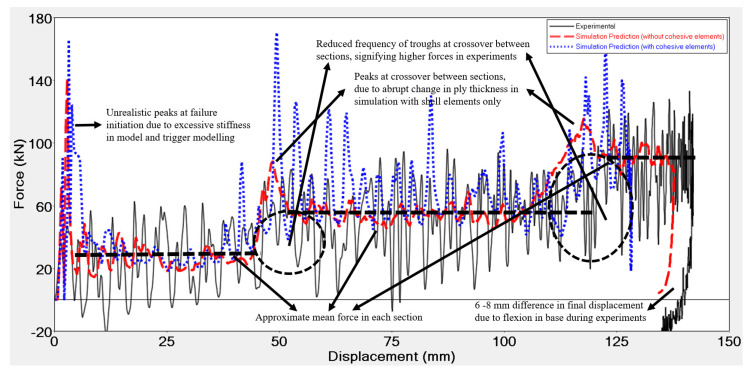
Comparison of force vs. displacement curves for experimental test and numerical simulations with and without cohesive elements on a Formula SAE impact attenuator impacted at 7300 J with an impact velocity of 7 m/s.

Using the optimized material card, simulations were run on the impact attenuator to validate the methodology and the results of the same are shown in Figure 13.

An initial peak, also seen in flat plate tests, was observed in the impact attenuator numerical simulation for both models, with and without cohesive elements. The peak in the flat plate tests was attributable to the difference in modelling the trigger as compared to its geometry in reality, but this was not the case with the impact attenuator. No trigger was embedded into the geometry of the impact attenuator as its increasing area and difference in the thicknesses of the three sections act as a trigger. The same was true for the numerical model. However, embedding a trigger to initiate failure could have reduced the peak in the numerical model. The high peak was partially, also, due to the numerical model of the base that was classified as a rigid body, which does not permit any DoFs ultimately making the model stiffer than it actually should be when compared with the physical setup wherein the base flexed by about 6–8 mm upon impact. Modelling the base with all DoFs without the rigid body, would have caused it to flex as it did during experimental testing, thereby reducing the initial peak and improving the correlation with experimental results. Subsequently, upon adding this displacement caused by the flexion of the base in the experiments to the final numerical displacement of the model without and with cohesive elements, the difference in the final displacement reduces to less than 5% and around 5%, respectively, for the two different adopted models. Displacement of model with cohesive elements was lower when compared to the model without cohesive elements because of higher forces in the middle section. Average crushing force for the three sections was predicted accurately for the model without cohesive elements. The same was not true for the model with cohesive elements, wherein the force was predicted accurately for the first sections, but was overestimated by about 10% for the middle section of the attenuator. This overestimation was possibly due to the higher value for the parameter $W_{p}$ obtained from the material model identification runs, which did not allow enough elements to be deleted and caused the walls to fall into the cavity of the attenuator. As the walls buckled without breaking, more elements stayed in contact with the impactor instead of detaching, thus leading to a higher force. The models overestimated the force at the interfaces between the sections. Although higher forces at troughs at the interface suggest that the experimental force there was higher as compared to the mean crush in the section before and after the interface, numerical simulation still over predicted the force in the interface. This over prediction was possibly due to an abrupt change in the thickness between the sections in the numerical model. In the physical impactor, however, the change may have been more gradual due to a manufacturing process that may have led to excess CFRP material depositing at these interfaces. Gradually increasing the thickness at the interfaces in the numerical model would have improved the correlation between the experimental and numerical results by reducing the peaks. Additionally, the using a finer mesh at the interfaces could serve the same purpose. Table 9 shows a comparison of run time between the different approaches for both the flat plate and crash box models.

Time step for the model with cohesive elements dropped by five orders of magnitude, due to compression of cohesive elements, from its initial time step, which could have caused the model to take an excess of 10,000 h to run. Therefore, a command was added to the model with shell and cohesive elements that disallowed the time step to drop below 66% of the initial time step. The command used has no effect on the results when used for models with non-hyperelastic materials and allowable time step less than the initial time step [52].

**Figure 14:**
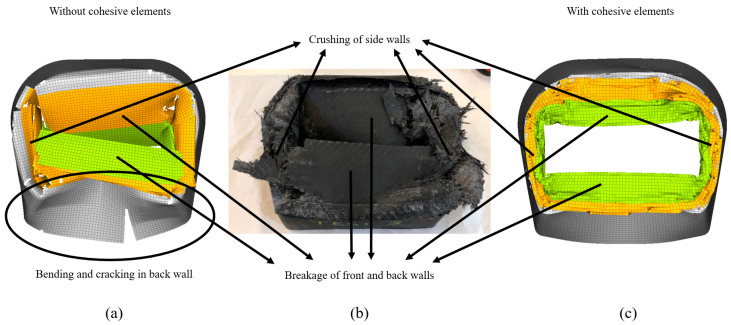
Comparison of final damage between attenuator models without cohesive elements (**a**) and with cohesive elements (**c**) and physical attenuator (**b**).

Figure 14 shows the final damage comparison between the experimental attenuator and the models with and without cohesive elements. Both the models were able to capture breakage of the front and back walls accurately. However, the model with cohesive elements better visualized sidewall crushing because introduction of cohesive elements made the model less stiff as cohesive elements resulted in bonding between plies that could be damaged allowing the plies to interact with each other as compared to perfect bonding between the plies when a single shell element represents four plies. As the meso-scale model was less brittle, local buckling was significantly reduced and was comparable to local buckling observed in the experiments, which was not the case with the macro-scale mode, wherein high local buckling led to cracking at the bottom. Another reason for the increased stiffness in the model was rigid modelling of the base plate, which did not flex as it did in the experiments.

**Figure 15:**
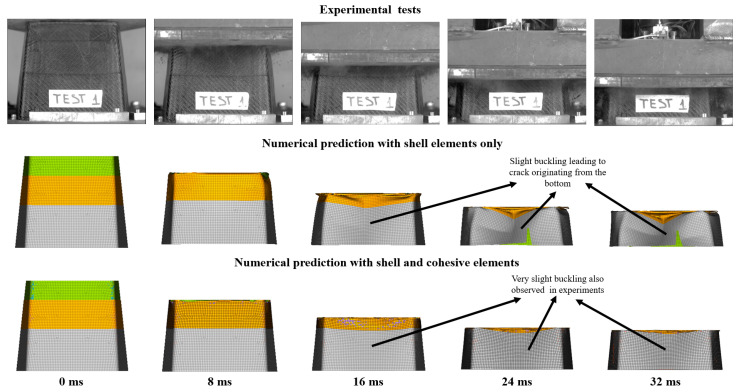
Time-lapse comparison of experimental results with numerical prediction results using both the macro- and meso-scale approach (front view).

Figure 15 shows a time-lapse comparison of the experimental results with numerical prediction results using both the macro- and meso-scale approach when viewed from the front. Buckling and the development of the crack using the macro-scale approach is seen more clearly. As mentioned earlier, the buckling observed in the meso-scale model was not significant and was comparable to that observed in the experiments. In general, the model without cohesive elements accurately predicted the behavior of the impact attenuator under impact loading and was able to model the damage visualization with reasonable accuracy. Additionally, the run time for the model without cohesive elements was significantly lower as compared to that with cohesive elements.

## 4. Conclusions

In this study, a methodology that is able to predict the impact response of composite components was presented. The methodology involved conducting in-plane impact tests on flat composite plates using a newly developed fixture that works seamlessly with Instron drop towers. The results of the experimental investigation on these flat plates were used to calibrate three numerical parameters of the material card in Radioss using an automated parametric identification procedure. The material card obtained was able to predict the impact response of a Formula SAE crash box made of the same material with reasonable accuracy for the crushing force values and history and for damage visualization. Stroke displacement was predicted within 5% of the experimental values, thereby validating the methodology. 

For the in-plane impact test, contact was best modelled when the contact stiffness applied to the contact between the elements of the composite specimen (self-contact) was set the minimum stiffness of 1 kN/mm. The contact stiffnesses for the rest of the contacts was calculated based on the Young’s modulus and thickness for the respective components. Comparison of the results obtained with the two considered optimization algorithms, GRSM and ARSM, showed that GRSM is more robust, and it is recommended for research purposes, whilst ARSM is more efficient, and can be used in the industry applications. The integral of the absolute difference between the numerical and experimental force values over the displacement was a better response objective as compared to squared difference, as the application of the former produces final stroke displacement values closer to those obtained experimentally. Identifications were able to obtain a material card that predicted both the force and stroke displacement simulated values within 5% of the experimental results. 

Although the macro model consisting only of shell elements was not able to capture delamination behavior, it was able to predict force and displacement of component upon impact with < 5% error, whilst simulating macro damage visualization with reasonable accuracy. The meso-scale model consisting of shell and cohesive elements captured delamination behavior accurately, but overestimated the force in the middle section by an acceptable 10%, in addition to taking significantly longer time to run. Therefore, a macro-scale approach is suggested for industrial applications. Overestimation of force due to non-deletion of elements in the meso-scale approach as a consequence of the high values obtained for the primary element deletion criterion could be resolved by using additional failure criteria. 

The developed methodology should help increased integration of composite materials into primary crash structures due to reduced expenditure on expensive experimental tests as only material characterization and crashworthiness tests using the anti-buckling fixture will need to be conducted, whilst component and parts of full-scale testing could be replaced by numerical analysis. This would also result in relevant timesaving, thereby leading to reduced costs for development of such structures. The adoption of composite material structures should ultimately lead to lower emissions of noxious gases for vehicles, as they would be significantly lighter taking advantage of the higher SEA of composite materials as compared to metals.

## Figures and Tables

**Table 1:** Material characterization data for carbon fibre reinforced polymer (CFRP) material used.

Property	GG630T-37 Carbon Fibre Laminate [28]
Elastic modulus (GPa)	60 ± 2.21
Tensile strength (MPa)	946 ± 37.36
Flexural strength (MPa)	624 ± 48.05
Compressive strength (MPa)	325 ± 13.03

**Table 2:** Dynamic tests conditions at the drop tower facility in Picchio Spa. with and impact mass of 300 kg.

Test Number	Impact Velocity (m/s)	Impact Energy (J)
1	7.10	7561
2	7.10	7561
3	7.77	9055
4	8.04	9696

**Table 3:** Material properties of C40 steel [29].

Parameter	Value	Parameter	Value
Density (g/cm^3^)	7.85	Hardening Parameter	0.7
Young’s Modulus (GPa)	202	Hardening Exponent	0.4
Poisson’s Ratio	0.3	Failure Plastic Strain	0.16
Yield Stress (MPa)	230	Maximum Stress (MPa)	560

**Table 4:** Material properties of CFRP specimen.

Parameter	Value	Parameter	Value
Density (g/cm^3^)	1.56	Shear Yield Strength (MPa)	10
Young’s Modulus (GPa)	70	Ult. Shear Strength (MPa)	65
Poisson’s Ratio	0.075	Failure Strain	0.018084
Shear Modulus 12 (GPa)	4	Energy Failure Value * (J/mm^3^)	0.0846
Ult. Tensile Strength (MPa)	911	Compressive Residual Stress * (MPa)	132
Ult. Compressive Strength (MPa)	334	Shear Residual Stress *(MPa)	34

* obtained from numerical identification (all the other values were obtained from experiments).

**Table 5:** Relevant material properties of the adhesive layer modelled using cohesive elements.

Parameter	Value	Parameter	Value
Young’s Modulus (GPa)	3.2	Compression Modulus (MPa)	8
Shear Modulus (GPa)	2	Yield Stress (MPa)	75
Failure Strain	0.045	–	–

**Table 6:** Specific energy absorption calculated for flat plate tests conducted with Instron 9450 drop tower using an impact mass of 50 kg and different impact velocities.

Material	Impact Velocity (m/s)	Impact Mass (kg)	Impact Energy (J)	SEA (kJ/kg)	Std.
GG630T-37 carbon fibre laminate [28]	4.69	34	375	45.537	1.30
	4.69	50	550	46.532	2.73
	4.69	70	770	45.945	2.96
	5.29	50	700	45.350	2.46
	5.83	50	850	45.002	1.83

**Table 7:** Comparison of different contact stiffness formulation combinations on damage behavior and overview of the cases selected to be optimized.

Case	Stiffness Formulation	Deformation Type	Identification
A	Minimum stiffness for all contacts	Fronding at the bottom with delamination on top	
B	Calculated stiffness between supports and specimen	Fronding at the bottom with delamination on top	
C	Calculated stiffness between impactor, base and specimen	Fronding and local buckling at the bottom	
D	Calculated stiffness between elements of the specimen	Fronding at the bottom	
E	Calculated stiffness for all contacts	Fronding at the bottom	✔
F	Calculated stiffness between supports and specimen and elements of the specimen	Fronding at the bottom	✔
G	Calculated stiffness between impactor, base and specimen and supports and specimen	Fronding and local buckling at the bottom	✔
H	Calculated stiffness between impactor, base and specimen and elements of the specimen	Fronding at the bottom	

**Table 8:** Optimized values for both shell and shell + cohesive models.

Parameter	Shell Model Optimized Value	Shell + Cohesive Model Optimized Value
$W_{p}$ (J/mm^3^)	0.0846	0.4070
Compressive Residual Stress (MPa)	132	187
Shear Residual Stress (MPa)	34	39

**Table 9:** Run time comparisons between different approaches for flat plate and crash box.

Model	Modelling Type	Time
Flat plate	Shell only	5 min
	Shell + cohesive	65 min
Impact attenuator	Shell only	80 min
	Shell + cohesive	>10,000 h (extrapolated)
	Shell + cohesive (with time step limit)	80 h
